# Assessing the lung cancer risk reduction potential of candidate modified risk tobacco products

**DOI:** 10.1007/s11739-019-02045-z

**Published:** 2019-02-14

**Authors:** Julia Hoeng, Serge Maeder, Patrick Vanscheeuwijck, Manuel C. Peitsch

**Affiliations:** PMI R&D, Philip Morris Products S.A., Quai Jeanrenaud 5, 2000 Neuchâtel, Switzerland

**Keywords:** Candidate modified risk tobacco products, Non-clinical and clinical assessment, Lung cancer, Genetic damage, Inflammation

## Abstract

Smoking is the major cause of lung cancer. While the risk of lung cancer increases with the number of cigarettes smoked and the duration of smoking, it also decreases upon smoking cessation. The development of candidate modified risk tobacco products (cMRTP) is aimed at providing smokers who will not quit with alternatives to cigarettes that present less risk of harm and smoking-related disease. It is necessary to assess the risk reduction potential of cMRTPs, including their potential to reduce the risk of lung cancer. Assessing the lung cancer risk reduction potential of cMRTPs is hampered by (i) the absence of clinical risk markers that are predictive of future lung cancer development, (ii) the latency of lung cancer manifestation (decades of smoking), and (iii) the slow reduction in excess risk upon cessation and *a fortiori* upon switching to a cMRTP. It is, therefore, likely that only long-term epidemiology will provide definitive answers to this question and allow to first verify that a cMRTP reduces the risk of lung cancer and if it does, to quantify the reduction in excess lung cancer risk associated with a cMRTP. For this to be possible, the cMRTP would need to be available in the market and used exclusively by a large portion of current smokers. Here, we propose that a mechanism-based approach represents a solid alternative to show in a pre-market setting that switching to a cMRTP is likely to significantly reduce the risk of lung cancer. This approach is based on the causal chain of events that leads from smoking to disease and leverages both non-clinical and clinical studies as well as the principles of systems toxicology. We also discuss several important challenges inherent to the assessment of cMRTPs as well as key aspects regarding product use behavior.

## Introduction

Cigarette smoking is one of the leading preventable causes of human morbidity and mortality, causing serious diseases such as cardiovascular diseases (CVD), chronic obstructive pulmonary disease (COPD), and lung cancer. The vast majority of smoking-related diseases are caused by the toxicants^1^ present in cigarette smoke, which are mostly formed during the combustion of tobacco.^2^ The U.S. Surgeon General has stated that the “burden of death and disease from tobacco use in the United States is overwhelmingly caused by cigarettes and other combusted tobacco products” [1]. Nicotine, while addictive, not risk-free, and an important factor of why people smoke, is not the primary cause of diseases [2].

For decades, the efforts to reduce the harm caused by smoking have been focused on preventing smoking initiation and promoting smoking cessation [3, 4]. More recently, Tobacco Harm Reduction (THR) has emerged as a third and complementary approach that can help to reduce the adverse effects of smoking [5]. Modern THR is based on switching consumers to less harmful products that emit significantly lower levels of toxicants, while providing levels of nicotine comparable to cigarettes [2, 6]. As noted by McNeil [7], “Since nicotine itself is not a highly hazardous drug, encouraging smokers to obtain nicotine from sources that do not involve tobacco combustion is a potential means to reduce the morbidity and mortality they sustain, without the need to overcome their addiction to nicotine.” This new approach complements those aimed at reducing smoking prevalence and aims to provide smokers who will not quit with novel tobacco or nicotine-containing products that are substantially less toxic than cigarettes. The U.S. Family Smoking Prevention and Tobacco Control Act (FSPTCA) embraces the concept of THR and defines a modified risk tobacco product (MRTP) as any tobacco product that is sold or distributed for use to reduce harm or the risk of tobacco-related disease associated with commercially marketed tobacco products [8].

Candidate MRTPs (cMRTP) are products designed to avoid combustion and thereby significantly reduce the emission of toxicants while delivering satisfying levels of nicotine, sensory satisfaction, and a ritual close to that of cigarettes. cMRTPs that deliver nicotine-containing aerosol are based primarily on two technologies. First, electronic cigarettes (e-cigarettes) generate the aerosol from a flavored e-liquid with a heating element consisting of a coil around a wick. Generally, e-liquids are mixtures of constituents such as vegetable glycerin (VG), propylene glycol (PG), water, nicotine, and flavors. Second, heated tobacco products heat a tobacco substrate, at temperatures well below that needed for combustion, using either a carbon-based heat source or an electronically controlled heating element. This leads to the formation of an aerosol consisting mainly of the water, VG, nicotine, and flavors contained in the tobacco substrate. Both technologies deliver various levels of nicotine as well as low levels of certain toxicants due to the limited thermal degradation of the heated substrate. Therefore, MRTPs will not be risk free.

A cMRTP can be authorized by the U.S. Food and Drug Administration (FDA) as an MRTP under the FSPTCA if the product, as actually used, will (Part A) significantly reduce harm and the risk of tobacco-related disease to individual tobacco users, and (Part B) benefit the health of the population as a whole, taking into account both the users of tobacco products and persons who do not currently use tobacco products [9]. This means that a cMRTP may contribute positively to population harm reduction, if it is of significantly lower risk than cigarettes for the individual tobacco user and, a significant number of current adult smokers are willing to switch to the product. Because MRTPs are not risk free, this also means that the product should not attract persons who do not currently use tobacco products (i.e., never smokers or former smokers) and should be used in lieu of cigarettes and not in addition to cigarettes [6].

The main objective of this contribution is to describe a possible approach to lung cancer risk assessment that satisfies Part A mentioned above. Key aspects pertinent to Part B are highlighted in the "Product use behavior" section.

As described previously, there is a broad and evolving diversity of cMRTPs with different levels of emissions, and hence associated cancer potencies [10]. Therefore, it is important to assess each cMRTP for its harm and disease risk reduction potential, which is discussed in the *challenges* section. Nevertheless, the assessment should be conducted within a framework that is informed by the known epidemiology of smoking and cessation. In short, successful cMRTPs must have a risk profile that (i) is significantly lower than that of cigarettes and, (ii) approaches that of smoking cessation, which is the best possible option for smokers [6].

## From challenges to a possible approach to lung cancer risk assessment

Smoking is the major cause of lung cancer, and this risk increases with the number of cigarettes smoked and the duration of smoking [3, 11]. It is also known that the risk of lung cancer decreases upon cessation [12, 13], with a slow decline in excess risk (approx. 50% excess risk reduction 10 years after quitting) [14]. This decrease in lung cancer risk upon smoking cessation is due to the discontinuation of the exposure to the toxicants contained in cigarette smoke.

Assessing the lung cancer risk reduction potential of cMRTPs before or soon after they are introduced into the market is hampered by (i) the absence of clinical risk markers that are predictive of future lung cancer development, (ii) the latency of lung cancer manifestation (decades of smoking), and (iii) the slow reduction in excess risk upon smoking cessation and *a fortiori* upon switching to a cMRTP. It is, therefore, likely that only long-term epidemiology will provide definitive answers to this crucial question and allow to (a) verify that a cMRTP reduces the risk of lung cancer and (b) if it does, to quantify the reduction in excess lung cancer risk associated with a cMRTP. For this to be possible, the cMRTP would need to be available in the market and used exclusively by a large portion of current smokers. In this context, we propose that a mechanism-based approach represents a solid alternative to show that switching to a cMRTP is likely to significantly reduce the risk of lung cancer in a pre-market setting. This approach is based on the previously described causal chain of events that leads from smoking to disease manifestation [6] and leverages the principles of systems toxicology [15].

Smoking-related lung cancer is caused by chronic exposure to the carcinogenic toxicants found in tobacco smoke. These substances trigger the key pathways that lead to cancer. Carcinogenic toxicants contained in cigarette smoke, such as TSNAs, metabolites of polycyclic aromatic hydrocarbons (PAH), free radicals [including reactive oxygen species (ROS) and reactive nitrogen species (RNS)], and various aldehydes, will cause genetic damage that can lead to the loss of normal cellular growth control mechanisms and cell proliferation [16–18]. The toxicants in cigarette smoke also cause chronic inflammation [19–21], which promotes tumor formation [22, 23]. The 2010 U.S. Surgeon General’s Report, *How Tobacco Smoke Causes Disease: The Biology and Behavioral Basis for Smoking*-*Attributable Disease*, identifies inflammation and oxidative stress, among others, as key mechanisms underlying all major smoking-related diseases [3].

**Fig. 1:**
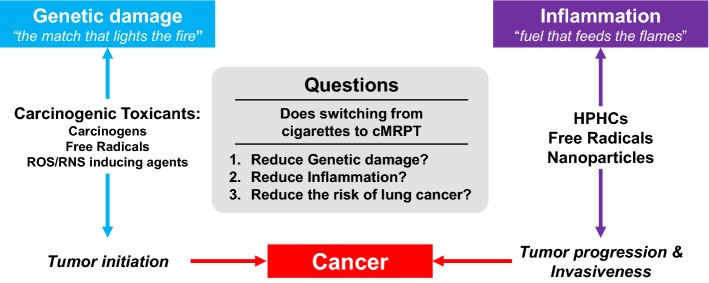
Approach to lung cancer based on the hypothesis of Balkwill and Mantonvani: “if *genetic damage* is the match that lights the fire of cancer, some types of *inflammation* may provide the fuel that feeds the flames”. Carcinogenic toxicants include carcinogens, free radicals as well as ROS/RNS inducing agents. *cMRTP* candidate modified risk tobacco product, *HPHC* harmful and potentially harmful constituents

Balkwill and Mantovani hypothesized that “if *genetic damage* is the match that lights the fire of cancer, some types of *inflammation* may provide the fuel that feeds the flames” [24]. This hypothesis is consistent with the two characteristics, “genome instability and mutation” and “tumor-promoting inflammation”, that enable the acquisition of the hallmarks of cancer defined by Hanahan and Weinberg [25]. This provides us with a framework to develop an approach to assess cMRTPs for their potential to reduce the risk of lung cancer. This approach is based on three important questions that can be answered prior to cMRTP market introduction using a combination of non-clinical and clinical studies (Fig. 1):

- Does switching from cigarettes to the cMRTP reduce genetic damage?
- Does switching from cigarettes to the cMRTP reduce inflammation?
- Does switching from cigarettes to the cMRTP reduce the risk of lung cancer?

Based on the hypothesis of Balkwill and Mantovani, a positive answer to the first two questions should lead to a positive answer to the third question. A positive answer to all three questions would indicate that it is reasonably likely that switching from cigarette smoking to cMRTP use would reduce the risk of lung cancer.

## The approach

The proposed approach is based on answering the three questions formulated in the previous section through scientific evidence showing that a cMRTP aerosol has a significantly reduced effect on the discrete causal events that link smoking to disease compared with cigarette smoke [6]. These steps focus on the detailed analysis of (i) the cMRTP aerosol composition, (ii) the exposure of human subjects to toxicants, and (iii) the effects of the aerosol on biological mechanisms associated with toxicity and disease in laboratory and clinical studies [6].

### Question 1: Does switching from cigarettes to a cMRTP reduce genetic damage?

Cigarette smoke contains many carcinogenic toxicants, which includes carcinogens, free radicals as well as ROS/RNS inducing agents. Cigarette smoking leads to the uptake of these carcinogenic toxicants. Many of these carcinogenic toxicants can bind DNA directly, or after activation through enzymatic pathways [26], to form DNA adducts. DNA can also be altered by oxidative damage induced by free radicals, such as ROS and RNS contained in cigarette smoke, or formed endogenously by cells exposed to cigarette smoke constituents [27–30]. DNA damage triggers complex surveillance and repair systems of the cell [17]. Because this repair system is not error free, DNA strand breaks and erroneous base substitutions may occur and accumulate, eventually leading to genomic instability. In most cases, these errors lead to dysfunctional cells or programmed cell death. However, in some cases, this can lead to activating mutations in oncogenes, growth factors, and their receptors, or inactivating mutations in tumor suppressors, leading to changes in cellular function. These changes can generate neoplastic cell populations with the potential to form tumors, for instance, in a tumor-promoting chronically inflamed tissue environment [16, 31].

#### The causal chain of events linking smoking to genetic damage

To answer the first question, let us consider the causal chain of events linking smoking to genetic damage:

“Emission of carcinogenic toxicants” *leads to* “exposure to carcinogenic toxicants”, which in turn *leads to* “metabolic responses to toxicant exposure, genotoxicity, and DNA damage”.

A reduction in emission of carcinogenic toxicants should, therefore, lead to a reduction in exposure to these toxicants, a reduction in DNA damage and genotoxicity, and a reduction in the metabolic responses to this exposure.

**Fig. 2:**
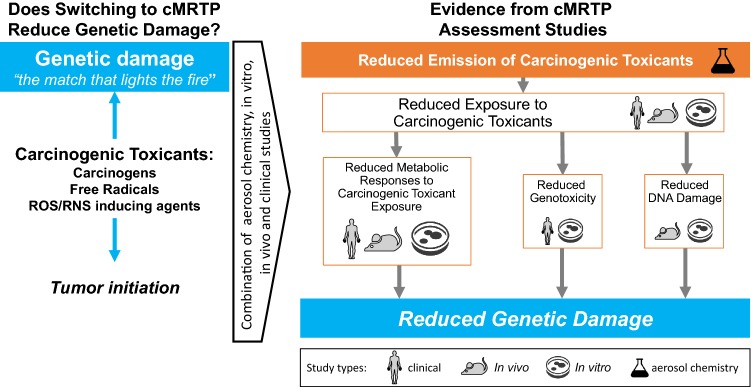
Does switching from cigarettes to a cMRTP reduce genetic damage? A combination of clinical, animal in vivo, human in vitro, and aerosol chemistry studies can provide the necessary evidence to answer this question. Carcinogenic toxicants include carcinogens, free radicals as well as ROS/RNS inducing agents. *cMRTP* candidate modified risk tobacco product

To gather evidence for each step in this causal chain of events, a combination of aerosol chemistry, non-clinical studies, and clinical studies can be used (Fig. 2):

1. *Emission of carcinogenic toxicants* The premise for a cMRTP to cause less genetic damage than cigarettes is that it must emit significantly lower levels of carcinogenic toxicants than cigarettes. The emission of both carcinogenic and noncarcinogenic harmful and potentially harmful constituents (HPHC) can be measured in both cMRTP aerosol and cigarette smoke with analytical chemistry methods. The levels of HPHCs [32–34] emitted by a cMRTP can thereby be compared with those emitted by a reference cigarette such as 3R4F from the University of Kentucky. In the context of cancer, one should not only focus on the reduction of the known carcinogens contained in cigarette smoke, but also ascertain that the emission of free radicals (including ROS and RNS) is reduced [35].
2. *Exposure to carcinogenic toxicants* A significant reduction in carcinogenic toxicant emission should then lead to a significant reduction in exposure to these substances in human subjects who switch from cigarette smoking to using a cMRTP in clinical studies. Ideally, these levels of exposure reduction should approach the levels of reduction observed in study subjects who abstained from smoking for the duration of the study [36–39]. As a direct consequence of a reduction in exposure to carcinogenic toxicants, switching to a cMRTP use should also lead to a significant reduction in urinary genotoxicity (a biomarker of total genotoxic exposure) compared with continued smoking and approach the urinary genotoxicity observed upon smoking abstinence [37]. The reduction in exposure to carcinogenic toxicants should also be assessed in both in vitro [40] and in vivo studies [41], an important step for quality control and interpretation of non-clinical studies.
3. *Metabolic responses to carcinogenic toxicants exposure* A significant reduction in exposure to carcinogens (including PAHs), free radicals (including ROS and RNS) and other oxidative stressors (such as aldehydes) should lead to a significant reduction in the activation of xenobiotic metabolism and oxidative stress responses.
  The activation and/or upregulation of many xenobiotic metabolism enzymes is driven by exposure to chemicals that can serve as substrates. The PAHs contained in smoke, such as benzo[a]pyrene, drive the upregulation of enzymes belonging to the Cytochrome P450 1A (CYP1A) and 1B (CYP1B) families. Therefore, members of these families are useful markers of exposure response. In particular, CYP1A2, which is important for the elimination of environmental chemicals, is strongly induced by PAHs. A reduction in PAH exposure should, therefore, lead to a reduction in CYP1A and CYP1B gene expression, protein abundance and enzymatic activity.
  Oxidative stress results from an imbalance between levels of oxidants and antioxidants. This imbalance allows ROS and other reactive species contained in cigarette smoke, or generated endogenously, to act directly on cellular components, damaging lipids, proteins and DNA. Glutathione (GSH) is a potent and essential intracellular free radical scavenging and oxidant detoxification agent. For instance, ROS oxidizes GSH to GSH disulfide (GSSG), while other HPHCs contained in cigarette smoke, such as acrolein [42], will bind to GSH via other mechanisms. Taken together, these oxidant detoxification mechanisms will lead to a depletion of the GSH pool, if the oxidant exposure is greater than the ability of the cell to produce and recycle oxidized GSH. In an attempt to counteract this depletion, cells exposed to cigarette smoke will increase the expression of enzymes involved in the biosynthesis of new GSH (GCLM and GCLC) and the recycling of GSSG to GSH (GSR). A reduction in exposure to free radicals and other oxidative stressors should, therefore, lead to a reduction in GSH depletion and a concomitant reduction in the expression of oxidative stress response genes, including those involved in maintaining the GSH pool.
  In clinical and in vivo studies, one can assess the effect of switching from cigarette smoking to cMRTP use on exposure responses by measuring biomarkers indicative of these mechanisms. For example, one can measure the plasma level of CYP1A2 enzyme activity, which should be reduced to a similar extent by switching from cigarette smoking to cMRTP use and smoking abstinence [37]. Similarly, exposure to cigarette smoke should induce both gene and protein expression of hepatic CYP1A2 in mice, while exposure to a cMRTP aerosol should not [43]. In both clinical and non-clinical in vivo studies, one can assess the level of oxidative stress by measuring urinary biomarkers such as 8-epi-Prostaglandine F2α [44], malondialdehyde or 4-hydroxynonenal [41]. These markers should also be reduced to a similar extent by switching from cigarette smoking to cMRTP use and smoking abstinence.
  Differences in xenobiotic metabolism and oxidative stress responses can also be assessed in human organotypic airway epithelial tissue cultures grown at the air–liquid interface. For instance, it has been shown that cigarette smoke exposure of these cultures recapitulates many of the gene expression changes induced by smoking in human airway biopsies [45]. In these cultures, cMRTP aerosol exposure should cause a reduced and more transient expression of xenobiotic metabolism (e.g., CYP1A1, CYP1B1, AKR1B/1C, ALDH3A1) and oxidative stress genes (e.g. NQO1, TXNRD1, GCLM/C, GSR, SRXN1) than cigarette smoke [40, 46].
  Furthermore, the aerosol of a cMRTP should have a reduced effect on the intracellular GSH pool and ROS generation compared to cigarette smoke. These toxicity endpoints can, for instance, be assessed in normal human bronchial epithelial cell cultures using High Content Screening [47, 48]. These types of studies can further yield useful gene expression measurements that can be used to compare the xenobiotic metabolism and oxidative stress responses induced by the aerosol of a cMRTP and cigarette smoke [42, 47].
4. *Genotoxicity* As a direct consequence of a reduction in carcinogenic toxicant emission, cMRTP aerosols should display significantly reduced mutagenicity and genotoxicity in standard cell-based assays, such as the Ames, in vitro micronucleus and the mouse lymphoma assays [32, 50].
5. *DNA damage* To confirm the reduced genotoxicity of the cMRTP aerosol, the biological response to DNA damage can be measured in vitro. For instance, the extent of DNA double-strand breaks is reflected by the increase in γH2AX, an early step in the DNA damage response (DDR) machinery [50]. An assay measuring the abundance of γH2AX in human bronchial epithelial cells can, therefore, be used to evaluate whether the extent of DNA double-strand breaks caused by the cMRTP aerosol is lower than that caused by cigarette smoke [47, 48]. Furthermore, in vitro and in vivo studies allow for the measurement of the relative expression levels of genes involved in the DDR machinery and provide the means to further confirm that a cMRTP aerosol causes less DNA damage than cigarette smoke in exposed tissues [40, 41, 46].

Taken together, a significant reduction in all steps of the causal chain of events linking smoking to genetic damage would demonstrate that a cMRTP causes less genetic damage than cigarette smoke (Fig. 2). This would indicate that the cMRTP aerosol is likely to cause less tumor initiation than cigarette smoke.

### Question 2: Does switching from cigarettes to a cMRTP reduce inflammation?

#### Inflammation and cancer

Many cancers arise in areas of chronic inflammation, which plays a major role in tumor invasion, progression, and metastasis, largely through the cytokine-mediated activation of mechanisms involved in tissue repair, cell proliferation and angiogenesis [20, 22, 51–53]. Furthermore, inflammation may also contribute to tumor initiation because activated inflammatory cells can induce the formation of ROS and RNS, which can induce genetic damage [20, 22]. Consequently, a reduction in inflammation should be accompanied by a reduction in cancer risk, which is confirmed by recent observations that reducing inflammation (e.g., through the chronic use of nonsteroidal anti-inflammatory drugs) can reduce the mortality of colorectal cancer and lung cancer [54–56]. Recent trials with anti-inflammatory treatments of COPD and CVD patients demonstrate an attenuation of the risk of developing lung cancer as a co-morbidity [57, 58].

Inflammation is of particular pathophysiological relevance to lung diseases and cancer, as chronic bronchitis triggered by asbestos, silica, smoke, and other inhaled toxins results in a persistent inflammatory response, which significantly increases the risk for lung cancer [59]. This is also supported by the fact that lung cancer is a frequent co-morbidity of COPD, a major inflammatory lung disease caused by smoke exposure. Indeed, COPD stages 1 and 2 and the presence of emphysema were shown to be among the strongest independent risk factors for lung cancer, with respective hazard ratios of 1.4 and 3.5 in the Pittsburgh Lung Screening Study cohort [23, 60]. This is not surprising, as COPD and lung cancer share several underlying disease mechanisms, such as oxidative stress and inflammation [59].

Smoke-induced lung inflammation is triggered by particulate matter and in part by aldehyde exposure, which leads to increased levels of, for example, interleukin 8 (IL-8) and monocyte chemoattractant protein 1 (MCP-1) in both mouse and human lungs [19, 61, 62]. It is also recognized that smoke exposure leads to the activation of the Nod-like receptor protein 3 (NLRP3) inflammasome [63], with consequent local generation of active interleukin 1β (IL-1β), a process that induces inflammation and thereby can lead to both chronic fibrosis and cancer in mice [64]. In 1996, Kuschner and colleagues showed that the concentrations of macrophages, neutrophils, IL-1β, and IL-8 are elevated in the pulmonary microenvironment of smokers in a dose-dependent manner [19]. It has long been known that in mice, inflammasome activation and IL-1β accelerate tumor invasiveness, growth, and metastatic spread [65]. For example, in IL-1β-deficient mice, neither local tumors nor lung metastases developed after localized or intravenous inoculation with melanoma cell lines, which suggests that IL-1β-induced inflammation participates in the invasiveness of already existing tumor cells [65]. This observation, made in an animal model, was recently confirmed by the results of the Canakinumab Anti-inflammatory Thrombosis Outcomes Study (CANTOS) [58]. Treatment with Canakinumab, a monoclonal antibody against IL-1β, led to a dose-dependent reduction in the concentrations of the inflammation markers high-sensitivity C-reactive protein and interleukin 6 (IL-6) as well as a reduction in lung cancer incidence and mortality. Taken together, these observations demonstrate that the chronic activation of the NLRP3 inflammasome, and the resulting inflammation, also play a role in lung cancer promotion.

#### Carbon-based nanoparticles

In addition to HPHCs and free radicals, incomplete combustion processes also generate solid carbon-based nano- and microparticles [66]. As the combustion of the tobacco is incomplete, cigarette smoke also contains solid carbon-based nanoparticles (cbNP) [67], which is consistent with the processes involved in soot formation during combustion [66]. These cbNPs consist, at least in part, of humic-like substances [67] and PAHs [68] surrounding a core of elemental carbon.

While HPHCs have been the main focus of cigarette smoke toxicity, it is important to also consider the health effects of the cbNPs generated during tobacco combustion and their contribution to lung injury. Numerous in vivo and in vitro studies have shown that nanoparticles, across a very wide range of sizes, shapes, and compositions, cause pulmonary inflammation [69, 70]. It has also been shown that human exposure to nanoscale carbon black leads to an increase in pro-inflammatory cytokines and a reduction in pulmonary function [71].

Lung exposure to fine particulate matter, such as monosodium urate crystals, asbestos, and crystalline silica, results in persistent inflammation, mediated primarily by the NLRP3 inflammasome [72]. Similarly, mouse lung exposure to carbon black nanoparticles causes the activation of the NLRP3 inflammasome, which leads to lung injury and emphysema [73]. This is consistent with previous observations that cbNP exposure leads to increased levels of IL-1β in mice [73] and humans [71]. It has also been shown that cbNPs accumulate in antigen-presenting dendritic cells derived from emphysematous lung tissue of smokers [73] and that cigarette smoke triggers the NLRP3 inflammasome [63], which is again consistent with the observation that IL-1β levels are elevated in smokers’ lungs [19].

Taken together, these lines of evidence suggest that cbNPs may participate in the pro-inflammatory effect of cigarette smoke.

#### The causal chain of events linking smoking to inflammation

To answer the second question, let us consider the causal chain of events linking smoking to lung inflammation:

“Emission of toxicants” *leads to* “exposure to toxicants”, which in turn *leads to* “lung inflammation”

A reduction in toxicant emission should, therefore, lead to a reduction in exposure to toxicants, which in turn should lead to a reduction in lung inflammation.

**Fig. 3:**
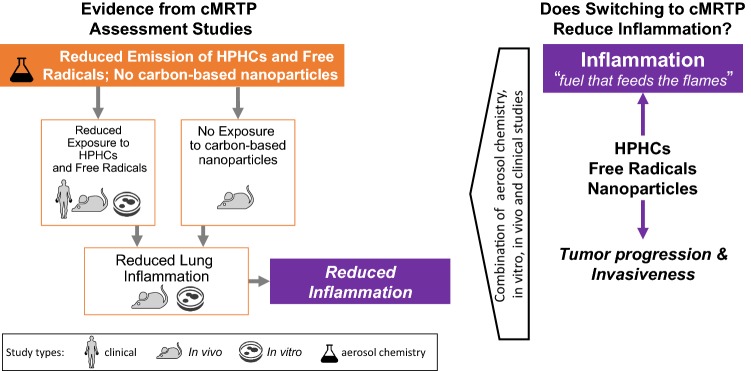
Does switching from cigarettes to a cMRTP reduce inflammation? A combination of clinical, animal in vivo, human in vitro, and aerosol chemistry studies can provide the necessary evidence to answer this question. *cMRTP* candidate modified risk tobacco product, *HPHC* harmful and potentially harmful constituents

A combination of several lines of evidence can be used to answer the second question (Fig. 3):

1. *Emission of toxicants* As outlined in the previous section, cMRTPs emit significantly lower levels of both carcinogenic and noncarcinogenic HPHCs [32–35], including aldehydes that are known to trigger inflammation (e.g., release of IL-8 and MCP-1) [61, 62]. Importantly, cMRTP aerosols should consist only of liquid droplets and contain little to no cbNPs due to the absence of combustion in these products. To verify this point, we have recently developed a method to quantify the level of cbNPs in cigarette smoke and cMRTP aerosols. The method requires stripping the smoke or aerosol of its volatile constituents, in other terms the liquid phase of the aerosol particles, before quantifying the remaining solid particles. This was achieved by passing the aerosol and the smoke through a commercially available Dekati^®^ Thermodenuder operating at 300 °C [74]. The analysis of the materials collected during this process by scanning electron microscopy revealed that the smoke of a 3R4F reference cigarette contained approximately 6 × 10^11^ ultra-fine solid particles of a median diameter of 75 nm (90% of the particles had a diameter between 20 and 120 nm) [74]. Taking into account the size distribution of these particles [74] and a carbon density corresponding to graphite (1.8–2.1 g/cm^3^), the calculated total mass of 6x10^11^ particles corresponds to 626–730 µg/cigarette. The fraction of particles between 20 and 100 nm in size corresponds to 291–340 µg/cigarette. These cbNPs were further determined to consist mainly of carbon-based materials, low amounts of oxygen, and traces of potassium, chlorine, aluminum, and silica [74]. In contrast, the analyzed cMRTP aerosol did not contain a measurable number of solid cbNPs [74]. Therefore, cMRTP aerosols, whether generated by heated tobacco products or e-cigarettes, should consist only of liquid droplets/particles. The toxicity of these liquid droplets/particles is a function of their chemical composition, i.e., the toxicants they contain.
2. *Exposure to toxicants* Similar to the reduced carcinogen exposure outlined in the previous section, a reduction in overall toxicant emission should lead to a significant reduction in toxicant exposure in both laboratory and clinical studies. While exposure levels to several noncarcinogenic HPHCs, such as carbon monoxide and acrolein, can be assessed in clinical studies, as outlined above, exposure to cbNPs cannot. Therefore, alternative assessment methods must be used. For instance, we have reported that cigarette smoke, but not the aerosol of a heated tobacco product, caused the discoloration of dental resin composites used for fillings as well as human dentin and enamel [75]. Furthermore, in a recent six-month e-liquid inhalation study conducted in mice, we observed that the lungs of mice exposed to cigarette smoke had a dark color, while those exposed to aerosol from an e-liquid were of the same light color as fresh air-exposed mice (Fig. 4). While there are many chromogenic substances in cigarette smoke, it is most likely that the discoloration of dental resin composites and mouse lungs is largely due to the deposition of cbNPs.

**Fig. 4:**
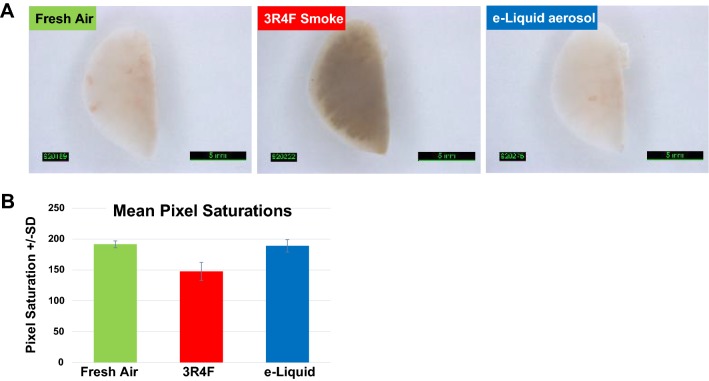
Lung discoloration. **a** Mice were exposed for 3 h/day, 5 days/week, for 6 months to either fresh air, smoke of the 3R4F reference cigarette, or aerosol of a flavored e-liquid composed of 66.7% PG, 28.5% VG, 4.8% nicotine, and 0.12% of a flavor mixture. The concentration of nicotine was 36.7 µg/L in the test atmosphere. Lungs were flushed with phosphate-buffered saline (PBS) to collect bronchoalveolar lavage fluid (BALF). Flushed left lungs were imaged under immersion in PBS. **b** Mean pixel saturations of the lungs determined using Visiopharm^®^ image analysis software; pixel saturations range from 0 (black) to 255 (white)
3. *Lung inflammation* These reductions in toxicant exposure should then lead to a reduction in lung inflammation. The extent of lung inflammation can be measured in studies conducted in animal models of disease. Such studies should confirm that cigarette smoke exposure causes lung inflammation (positive control), while cMRTP aerosols should have a very limited effect on this mechanism [41]. Furthermore, a study design that includes both a cMRTP switching and a cessation arm (exposure to cigarette smoke for a few months followed by exposure to either cMRTP aerosol or fresh air for several months) is essential to assess (i) the effects of a change in exposure that mimics the intended use of cMRTPs (i.e., switching current smokers) and (ii) how this compares with the effects of smoking cessation. In such studies, the extent of lung inflammation can be assessed by BALF analysis (quantification of inflammatory cells, such as neutrophils and macrophages, and molecular markers, such as cytokines and chemokines), histopathology (assessment of lung infiltration by inflammatory cells, such as neutrophils and pigmented macrophages), and both lung gene and protein expression analysis [41]. The effects of cigarette smoke and cMRTP aerosol on lung inflammation can also be assessed in vitro [40, 46] using both gene expression and protein abundance measurements.
  Several lung inflammation markers have been reported to be increased in smokers (neutrophils, macrophages, IL-1β, IL-8, IL-6, and MCP-1) [19]. It is of particular interest that these markers were also increased by exposure to cigarette smoke, but not by exposure to cMRTP aerosol, in a mouse model of disease [41]. Furthermore, switching from smoke exposure to either cMRTP aerosol or fresh air (to mimic smoking cessation) reduced the levels of these inflammatory markers [41]. This confirms not only that mouse models can be relevant to key aspects of human biology [58, 65], but also that the NLRP3 inflammasome is activated by smoke exposure in both mice and humans and may play a role in the inflammatory processes involved in lung tumor promotion [58, 65]. Furthermore, cigarette smoke also induced the expression of several inflammation markers, including IL-1β, in human organotypic airway epithelial tissue cultures that are grown at the air–liquid interface. In contrast, cMRTP aerosols applied at equivalent nicotine concentrations had little or no effect on these markers in these cultures [40, 46]. Finally, one can also assess the immune cell responses to cMRTP aerosol exposure using cell lines or primary cells and compare them with those caused by cigarette smoke. These studies show that immune cell responses are less affected by cMRTP aerosols than cigarette smoke, especially regarding their release of chemokines and cytokines such as IL-8 and TNFα [76, 77].
  In clinical studies, the effect of cigarette smoking on systemic inflammation can be assessed by measuring, for example, circulating white blood cell counts and C-reactive protein levels; while a direct quantification of lung inflammation is more difficult, as it involves a more invasive procedure. It has been suggested that small positive changes in lung function and respiratory symptoms, in smokers who switched to a cMRTP for 6–12 months, may be a reasonable proxy for a reduction in lung inflammation [78, 79]. This topic needs further research, especially to verify whether switching to a given cMRTP causes effects that approach those obtained by smoking cessation. But more generally, there is a need to identify other suitable lung inflammation markers that can be used in clinical studies.

Taken together, a significant reduction in all steps of the causal chains of events linking smoking to lung inflammation would demonstrate that a cMRTP causes less lung inflammation than cigarette smoke (Fig. 3). This would indicate that the cMRTP aerosol is likely to cause less tumor initiation, progression and invasiveness than cigarette smoke.

### Question 3: Does switching from cigarettes to a cMRTP reduce the risk of lung cancer?

cMRTP aerosols with significantly reduced effects on both key mechanisms involved in cancer causation hypothesized by Balkwill and Mantovani (*genetic damage* and *inflammation*) [24] would be reasonably expected to also reduce the risk of lung cancer compared with cigarette smoking. To confirm this, two complementary non-clinical approaches can be taken in the absence of long-term epidemiology.

First, in vitro studies can be conducted in cell lines to assess the relative effect of cigarette smoke and cMRTP aerosol on cellular and molecular endpoints linked to carcinogenesis. For instance, a Bhas 42 cell (mouse fibroblast-derived) transformation assay can be used to show that the aerosols of cMRTPs cause significantly less cell transformation than cigarette smoke [49]. Similarly, the long-term exposure (up to 12 weeks) of human bronchial epithelial BEAS-2B cells can be employed to show that the functional and molecular changes linked to lung carcinogenesis are less pronounced following cMRTP aerosol than cigarette smoke exposure [80].

Second, in vivo carcinogenesis studies, such as 18-month chronic inhalation studies, can be conducted in A/J mice to compare the effects of cMRTP aerosol and cigarette smoke on lung tumor incidence and multiplicity. The A/J mouse is highly susceptible to lung tumor development and has been widely used in carcinogenicity testing. These inbred mice often develop spontaneous benign tumors in the lung (adenomas) that may, on occasion, progress to cancerous lesions (adenocarcinomas). The A/J mouse strain is highly sensitive to toxicants/compounds that are carcinogenic, and exposure to these carcinogenic materials causes an increase in the number of animals that develop both adenomas and adenocarcinomas (incidence). In addition, a hallmark of carcinogen exposure in these mice is the occurrence of multiple lung tumors in any given animal (multiplicity) [81]. A/J mouse inhalation studies were carried out with cigarette smoke and showed that exposure to smoke leads to lung tumors [81]. While such studies are complex and require the use of mice, they enable a comprehensive non-clinical systems toxicology-based evaluation of all causally linked events linking smoking to disease [6, 15]. This means that in the same study, one can evaluate lung inflammation (BALF analysis, gene and protein expression analysis, histopathology), emphysematous changes (histopathology), lung function, and lung carcinogenesis (histopathology) together with carcinogen exposure (biomarkers of exposure, metabolic responses to carcinogen exposure [gene and protein expression]) and the response to DNA damage based on gene expression data. This allows for a coherent and integrated depiction of the biological effects of a cMRTP aerosol in comparison with those of cigarette smoke.

Taken together, a significant reduction in carcinogenesis endpoints in vitro and tumor formation in vivo would demonstrate that a cMRTP aerosol is less tumorigenic than cigarette smoke. In addition to a positive answer to both the first and the second question, this would indicate that smokers who switch from cigarette smoking to cMRTP use are likely to reduce their risk of lung cancer.

## Challenges

There are many challenges when implementing such an approach, beyond the already mentioned dearth of clinical risk markers for lung cancer and lung inflammation. The most important ones fall into these categories:

1. *Exposure of non-clinical test systems* non-clinical studies should be planned with two principles in mind. First, given that the aim of cMRTPs is to replace cigarettes, studies should always compare the effects of a cMRTP with those caused by cigarettes and, whenever possible, use smoking cessation as the benchmark as outlined previously [6]. Second, whenever possible, studies should cover a dose range, which includes a realistic human exposure dose. By combining these two principles, one can generally calculate (i) the dose at which a cMRTP aerosol has the same effect than cigarette smoke, (ii) their relative effects as a percentage, and (iii) the residual effect over cessation; importantly, these results can be put in the context of realistic human exposure levels. Achieving this can be challenging and great care has to be given when choosing exposure dose ranges, which can be based on (i) animal to human exposure conversion factors for in vivo studies [82] and (ii) human data for in vitro studies [83, 84].
2. *Non-clinical test systems* there is a need to develop more relevant human-derived in vitro systems to improve the assessment of cMRTPs and gradually replace in vivo rodent models. While the use of complex organotypic airway epithelium tissue cultures grown at the air–liquid interface is a step in the right direction, they are not representative of the alveoli and more generally lack the immune cells, fibroblasts and endothelial cells present in the lung. Consequently, improving the relevance of in vitro assay systems for the lung means developing co-cultures at the air–liquid interface that are able to mimic as closely as possible the physiological environment of the lung and the alveoli.
3. *Dealing with cMRTP diversity* numerous cMRTPs have been and will continue to be developed. They will likely differ in their toxicant emissions, which potentially lead to a broad range of product-specific toxicities [10]. Therefore, each cMRTP would have to undergo a complete assessment program as described previously [6]. It is, however, doubtful that this is a reasonable approach for the long term, mainly because of cost and time considerations. Therefore, a more pragmatic approach, that obtains both scientific and regulatory consensus, has to be developed. Key to this approach are (i) the use of agreed reference products for comparison, ideally authorized MRTPs, and (ii) bridging methodologies that allow to compare a cMRTP with the reference products based on a limited number of studies (see challenge 4).
4. *Bridging methodologies* to deal with the existing and future diversity of cMRTPs, and assess them for their risk reduction potential in a pragmatic manner, it will be necessary to develop bridging methodologies that enable a scientifically sound comparison of any cMRTP with a reference MRTP. Such methodologies should allow for a direct comparison of (i) toxicant emissions, (ii) a broad range of toxicity endpoints and (iii) reductions in toxicant exposure, to ensure that a given cMRTP is equivalent to, or better than, the reference MRTP. Such methodologies will, however, only address product characteristics, while product use patterns will need to be evaluated separately.

## Product use behavior

To fully assess the risk reduction potential of a cMRTP, one also has to evaluate how it will actually be used by consumers. To ensure that the market introduction of a cMRTP benefits the population as a whole, one has to take into account how the product will be used by smoker taking into account non-smokers [9]. Indeed, there is a legitimate concern that the availability of products with risk reduction claims may lead to an increase in overall nicotine product consumption in the general population. This risk can be assessed for each cMRTP.

First, a cMRTP should be attractive enough to adult smokers to encourage full switching, in other words completely replacing cigarette consumption by cMRTP use. Furthermore, the average level of daily nicotine consumption should not increase as a result of switching. These important parameters of a cMRTP can be assessed through measuring product use satisfaction, craving, nicotine exposure and daily consumption in clinical studies conducted in an ambulatory setting [44].

Second, the cMRTP should not attract non-smokers, including former smokers. The likelihood that a cMRTP attracts non-smokers should be evaluated prior to market introduction through perception and behavior assessment studies [8, 11]. Following market introduction, product use prevalence can be monitored through post-market studies [8].

Third, it is crucially important to carefully formulate the claims, labeling and communication materials associated with a cMRTP to ensure that the intended messages and warnings are accurate and not misleading [85], and well understood. These materials must be tested for comprehension in a representative sample of the population and must provide a clear understanding that (i) the best choice for a smoker is to quit, (ii) the cMRTP is not risk free, (iii) the cMRTP is addictive as it contains nicotine, and (iv) the cMRTP is not an alternative to quitting. Most importantly, cMRTPs, like any other tobacco product, should never be sold to underage individuals.

## Conclusions

Recently, an approach to determine the cancer potency and lifetime cancer risk associated with nicotine replacement therapy and a selection of cMRTPs was described [10]. This approach compares the cancer potencies of various cMRTP aerosols with those of cigarette smoke, nicotine inhalers, and ambient air. These cancer potencies are derived from the respective levels of carcinogen emissions, obtained through analytical chemistry, and their associated inhalation unit risks (i.e., the excess lifetime cancer risk from continuous inhalation exposure to a normalized concentration of the carcinogen). Lifetime cancer risks are then calculated from the cancer potencies using daily consumption estimates. While this approach provides a good initial evaluation of the risk reduction potential of a cMRTP, it should be complemented with studies conducted in living systems (such as those described above) to further strengthen the evidence regarding the lung cancer risk reduction potential of a cMRTP. One reason for this is that aerosols of cMRTPs are complex mixtures of both known and unknown constituents, most likely containing substances of unknown inhalation unit risk. A second reason is that one does not know whether these mixtures behave according to a linear combination of the effects of their individual constituents or whether synergistic effects occur.

All experimental biological systems have their shortcomings. Briefly, human in vitro systems do not reflect all aspects of cellular interaction and tissue organization, in vivo animal models do not necessarily reflect human biology in its finer details, and human clinical studies are hampered by time and cost constraints as well as accessibility to key biological samples (that are accessible in animal studies). Therefore, we have proposed an approach that integrates multiple lines of evidence derived from in vitro, in vivo, and clinical studies, enabling the shortcomings of each experimental system to be addressed with data derived from other systems to compare the effects of a cMRTP aerosol with those of cigarette smoke on the key steps of the causal chain of events linking smoking to disease. It is the totality of this evidence that should be considered when evaluating the risk reduction potential of a cMRTP.

The approach to the assessment of the lung cancer risk reduction potential of cMRTPs proposed here is based on the hypothesis of Balkwill and Mantovani [24]. The two key contributing pathways, *genetic damage* and *lung inflammation*, can be further subdivided into discrete, causally linked events, thereby providing a sound mechanistic basis for this assessment. Indeed, it is highly unlikely that a cMRTP which reduces the risk of lung cancer will not also reduce both *genetic damage* and *lung inflammation*, including all the steps in their respective causal chains of events. Likewise, it is highly unlikely that a cMRTP that does not emit substantially reduced levels of toxicants will significantly reduce both *genetic damage* and *lung inflammation,* and henceforth the risk of lung cancer. Therefore, we propose that this approach be recognized as a scientifically sound alternative to assess the risk reduction potential of cMRTPs long before epidemiological evidence becomes available.

Furthermore, it is important to remember that the impact of a cMRTP on population-level risk reduction will be maximized if smokers switch completely to the product (and abandon cigarettes), while their daily nicotine exposure remains stable or is reduced, and if the cMRTP does not attract non-smokers.

Finally, one should never forget and consistently communicate that (i) the best choice for a smoker is to quit, (ii) cMRTPs are not risk free, (iii) cMRTPs are addictive as they contain nicotine and, (iv) cMRTPs are not alternatives to quitting. Most importantly, it must be made very clear that cMRTPs, like any other tobacco product, should never be sold to underage individuals.

## Footnotes

1 General term including harmful and potentially harmful constituents, free radicals, and carbon-based nanoparticles.

2 While most toxicants are formed by combustion processes, tobacco-specific nitrosamines (TSNA) are released from the tobacco.
